# Reducing sample consumption for serial crystallography using acoustic drop ejection

**DOI:** 10.1107/S1600577519009329

**Published:** 2019-08-16

**Authors:** Bradley Davy, Danny Axford, John H. Beale, Agata Butryn, Peter Docker, Ali Ebrahim, Gabriel Leen, Allen M. Orville, Robin L. Owen, Pierre Aller

**Affiliations:** a Diamond Light Source, Harwell Science and Innovation Campus, Didcot, Oxfordshire OX11 0DE, UK; b School of Biological Sciences, University of Essex, Wivenhoe Park, Colchester, Essex CO4 3SQ, UK; c PolyPico Technologies Ltd, Unit 10, Airways Technology Park, Rathmacullig Wes, Ballygarvan, Cork T12 DY95, Ireland; d Department of Electronic and Computer Engineering, University of Limerick, Ireland; e Research Complex at Harwell, Rutherford Appleton Laboratory, Didcot OX11 0FA, UK

**Keywords:** serial crystallography, sample delivery, fixed targets, acoustic dispensing

## Abstract

Efficient sample delivery is an essential aspect of serial crystallography at both synchrotrons and X-ray free-electron lasers. Rastering fixed target chips through the X-ray beam is an efficient method for serial delivery from the perspectives of both sample consumption and beam time usage. Here, an approach for loading fixed targets using acoustic drop ejection is presented that does not compromise crystal quality, can reduce sample consumption by more than an order of magnitude and allows serial diffraction to be collected from a larger proportion of the crystals in the slurry.

## Abstract 2

A new approach for efficiently loading fixed targets for serial crystallography using acoustic dispensing is presented.

## 1. Introduction   

Serial femtosecond crystallography (SFX) at X-ray free-electron lasers (XFELs) has become an important facet of the crystallographers’ toolbox, allowing both time-resolved and ground-state measurements of X-ray sensitive samples (Schlichting, 2015 ▸). However, the opportunities that the high peak brilliance and femtosecond duration of XFEL pulses provide come with a challenge: the need to provide new samples at the repetition rate of the X-ray source or detector. Several approaches have been developed to meet this challenge ranging from liquid jets to high viscosity extruders, on-demand droplet injectors coupled to a tape drive, and fixed targets (Grünbein & Kovacs, 2019 ▸; Martiel *et al.*, 2019 ▸). This complementary range of delivery solutions means that an approach can be chosen and tailored to best suit the experiment at hand.

The success and impact of SFX has inspired the development and implementation of serial synchrotron crystallography (SSX), where many of the same sample delivery techniques are used (Diederichs & Wang, 2017 ▸). The subsequent success of SSX has now driven the development of synchrotron beamlines dedicated to serial crystallography, such as P14.EH2 at PETRA III (http://www.embl-hamburg.de/services/mx/P14_EH2/index.html), and this illustrates the desire of structural biologists to exploit serial approaches.

A challenge common to many serial approaches is sample consumption. The volume of sample consumed is many orders of magnitude greater than that required for traditional synchrotron approaches when a complete dataset may be obtained from a single crystal held at 100 K. Indeed, the sample requirements for a serial experiment are often an unwelcome surprise for the first-time user of SFX or SSX, as usually only a single ‘still’ image is collected from each crystal. This is reflected by developments to reduce sample con­sumption for serial experiments such as flow-focusing for in-flow SSX (Monteiro *et al.*, 2019 ▸).

Recently, we have developed fixed target sample delivery as a serial approach that works well at both synchrotrons and XFELs. These are based on thin films (Doak *et al.*, 2018 ▸) and, predominantly for us, silicon nitride ‘chips’ (Ebrahim *et al.*, 2019 ▸). Typically, to load a silicon nitride chip, ∼100–200 µl of crystal slurry is required. This slurry is pipetted over a chip and crystals are drawn to the apertures through use of a weak vacuum. Sufficient data for structure solution can typically be obtained from a single chip.

Acoustic dispensing is a technique that uses high-frequency acoustic waves to dispense small volumes of liquid. The ejected droplets may contain protein crystals (Soares *et al.*, 2011 ▸; Roessler *et al.*, 2016 ▸; Fuller *et al.*, 2017 ▸), live cells (Demirci & Montesano, 2007 ▸) or indeed almost any small molecule (Teplitsky *et al.*, 2015 ▸). Commonly referred to as acoustic drop ejection (ADE), here we use a variant that makes use of disposable dispensing cartridges allowing rapid switching between samples (Leen, 2016 ▸). Using the commercially available PolyPico pico-litre dispenser (https://www.polypico.com) synchronized with compact, high-precision *xyz* stages (http://www.smaract.com), we demonstrate the use of ADE to dramatically reduce sample consumption for fixed target serial crystallography.

## 2. Methods   

**Figure 1:**
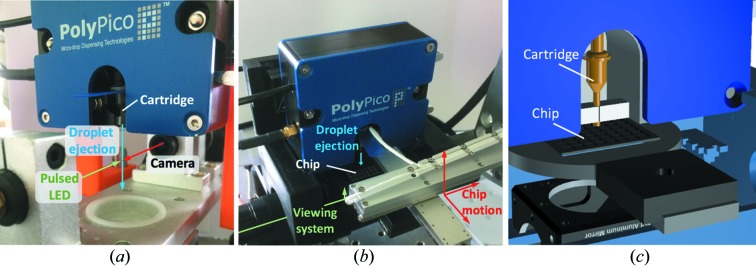
Experimental setup for (*a*) calibration of ejected droplets and (*b*) chip loading; in each, the direction of droplet ejection is shown in blue. (*c*) Schematic of chip loading from a similar viewpoint to (*b*) with translation stages hidden and the cartridge highlighted in yellow.

The loading of fixed targets using acoustic dispensing is a two-step process with a calibration step required prior to chip loading. For convenience we physically separate these steps [Figs. 1 ▸(*a*) and 1(*b*)]. The same PolyPico head dispenser was used for both aspects of the experiment and was mounted on kinematic mounts allowing transfer between calibration and loading in a few seconds.

### 2.1. Drop calibration   

For optimal loading of chips, the volume of droplets ejected by the PolyPico dispenser should be calibrated for each crystal slurry. Using a pipette and a tip-like adapter, the crystal slurry is loaded into a cartridge which has a dispensing aperture ranging from 30 µm to 150 µm in diameter. For the experiments described here, we loaded 10–20 µl of slurry into cartridges with any slurry not used easily recovered after the experiment using the same pipette and adapter. The cartridge aperture size is chosen based on the typical size of the crystals in the slurry. In practice, we find that an aperture diameter approximately twice the size of the crystals used works well as a compromise between minimizing drop size and avoiding clogging if larger crystals are present. The width, amplitude and frequency of the acoustic wave applied to the cartridge base must be tuned until stable droplets are ejected from the crystal slurry. Ejected droplets are visualized using a high-resolution camera and stroboscopic LED [Fig. 1 ▸(*a*)] with image recognition software allowing real-time readback of the average droplet volume. Typically, when using a 1 kHz acoustic wave and a cartridge aperture of 100 µm, 80–100 pl (approximate diameter 60 µm) droplets can be obtained. Once the optimal parameters for ADE of crystal slurry have been determined, chips can be loaded.

### 2.2. Chip loading   

The setup for ADE loading of fixed targets is shown in Figs. 1 ▸(*b*) and 1(*c*). Chips are mounted on a three-axis stage and can be viewed through a high-resolution camera which allows viewing of both fixed targets and droplets ejected by the dispensing head. The tip of the dispensing head is within 0.5 mm of the surface of the chip. Following alignment of chip fiducials, chips can be moved as previously described (Sherrell *et al.*, 2015 ▸). In this case the stages act as the ‘master’, sending a TTL pulse to the dispensing head with droplets ejected on demand when each aperture is reached. Following the ejection of a user-defined number of droplets at 1 kHz, the stages move to the next aperture on the chip. The loading of a chip with 25 600 positions takes less than 4 min and consumes less than 4 µl of slurry. To avoid dehydration, the chip and dispensing head are enclosed in a high-relative-humidity environment (>90%) [Fig. 1 ▸(*b*)]. Following loading, the chips are sealed with a thin film (typically 6 µm) of mylar. Chips with a funnel-shaped aperture (size of the small end of the funnel: 7 µm) were used; the volume of each aperture was ∼160 pl, and apertures are spaced by 125 µm (centre-of-aperture to centre-of-aperture distance).

In order to conserve sample and also minimize the beam time required for X-ray data collection, only the central area of chips was acoustically loaded (6 × 6 ‘city blocks’, 14 400 apertures) in the experiments described here. In this case, the time required to load a chip was 2 min 15 s. In total, the complete acoustic loading process including alignment and loading takes approximately 5 min (full chip), and throughput is equal to or faster than X-ray data collection. Chips were loaded by both using the ADE approach described above and also, for comparison, manually using a pipette.

### 2.3. Sample preparation   

Microcrystals of chicken egg-white lysozyme (HEWL) were prepared using an adaptation of a previously described protocol. In brief, high-purity lysozyme powder (Sigma–Aldrich L6876-5 G) was resuspended in 100 m*M* sodium acetate pH 3.0 to a final concentration of 25 mg ml^−1^ and mixed with an equal volume of crystallization buffer (16.8% *w*/*v* sodium chloride, 4.8% *w*/*v* PEG 6000 and 0.06 *M* sodium acetate pH 3.0) at room temperature. The mixture was vortexed for 10 s and then left for an hour until crystal growth saturation. Using this method, we obtained homogeneous rectangular crystals with an average size of 10 µm × 10 µm × 15 µm. Microcrystals of copper nitrite reductase from *Achromobacter cycloclastes* (*Ac*NiR) ranging in size from 15 µm to 70 µm were grown using a protocol described previously (Ebrahim *et al.*, 2019 ▸). The concentration of crystals in each slurry was estimated using a Hemocytometer cell counter.

### 2.4. X-ray data collection   

Following loading, chips were transferred to Beamline I24, Diamond Light Source. Diffraction data were collected as previously described (Owen *et al.*, 2017 ▸) though only from the central region of chips loaded by the PolyPico (14 400 apertures), with data collection taking 4 min 20 s. Data were collected using an X-ray energy of 12.8 keV, a beam size of 7 µm × 6 µm, 10 ms exposures and a flux attenuated to 8 × 10^11^ photons s^−1^.

Hit-rates were obtained using dials.stills_process (Winter *et al.*, 2018 ▸; Brewster *et al.*, 2016 ▸, 2018 ▸) with up to ten lattices per image indexed. Subsequent scaling and merging of data was performed using *PRIME* (Uervirojnangkoorn *et al.*, 2015 ▸). In all cases the majority of indexed images contained a single lattice with the percentage of single lattice images being 77% (HEWL, PolyPico loaded), 81% (HEWL, pipette loaded), 85% (*Ac*NiR PolyPico loaded) and 66% (*Ac*NiR, pipette loaded). In the following, we define the diffraction hit-rate as the total number of indexed patterns divided by the number of collected images.

## 3. Results   

In preparatory experiments, we varied and defined the optimal number of acoustically ejected droplets. When dispensing two drops per single chip aperture, we observed higher hit-rates than when using a single droplet. The dispensing of three or more drops overflowed the apertures resulting in excess liquid on the surface of the chip. Therefore, all of the results presented here were obtained using two droplets per aperture.

**Figure 2:**
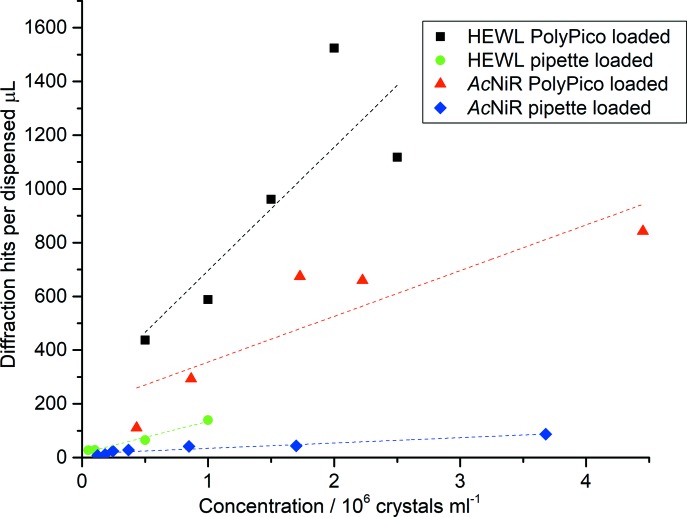
Diffraction hits per unit volume of dispensed crystal slurry. Acoustic dispensing results in more than a fivefold increase in hits per unit volume of slurry consumed at all concentrations for HEWL and a tenfold increase for *Ac*NiR.

Diffraction hit-rates for HEWL crystals loaded manually and using acoustic dispensing as a function of crystal concentration are shown in Table 1 ▸. As might be expected, in both cases diffraction hit-rates increase with crystal concentration. Also, for a given concentration, higher diffraction hit-rates are obtained using pipette loading. However, acoustic loading requires a significantly lower volume crystal slurry to achieve these, as illustrated by the number of diffraction hits obtained per dispensed microlitre of crystal slurry (Fig. 2 ▸).

Similar trends are seen for *Ac*NiR crystals (Fig. 2 ▸, Table 2 ▸), which significantly differ from HEWL crystals both in shape and chemical composition of the crystallization conditions, with an increasing hit-rate for both pipette and acoustically loaded chips as a function of slurry concentration. Higher diffraction hit-rates are also seen for pipette loaded chips at the expense of increased sample consumption.

Using the crystal concentration measured as described above, the number of crystals used in each experiment, and hence the fraction from which diffraction was recorded, can be estimated. We refer to this quantity as the absolute hit-rate and it is given for HEWL and *Ac*NiR in Tables 1 ▸ and 2 ▸, respectively. For pipette loading, it can be seen that, although higher diffraction hit-rates are achieved by increasing the crystal slurry concentration (this could also be achieved by simply increasing the volume of slurry loaded onto the chip), this is at the expense of the absolute hit-rate, with diffraction recorded from a decreasing proportion of the crystals used in the loading process. Although the diffraction hit-rate may be lower for acoustically loaded fixed targets, a larger proportion of the crystals grown produce a diffraction pattern.

**Figure 3:**
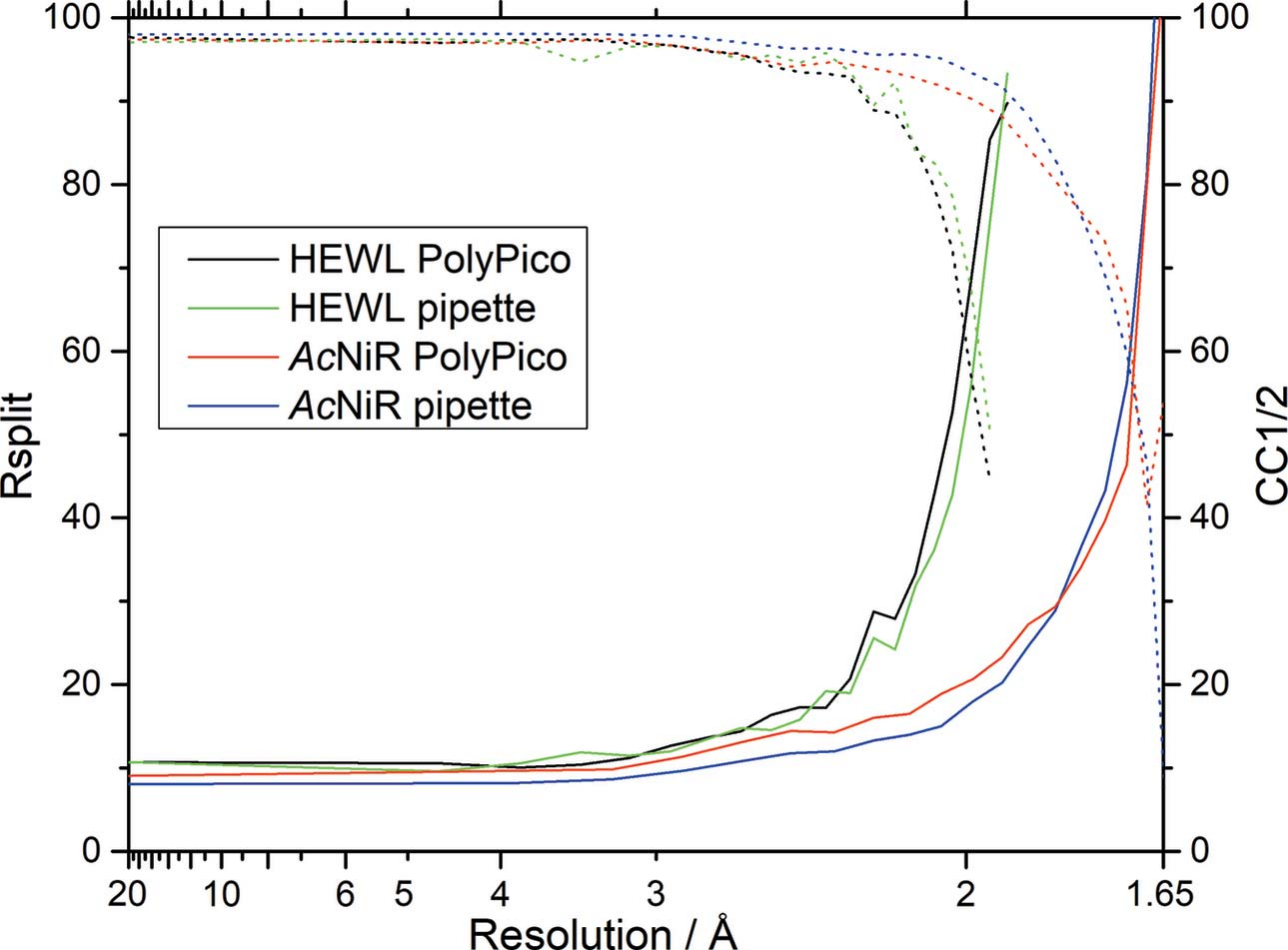
Data merging statistics. *R*_split_ (solid line) and CC_1/2_ (dotted line) are shown for HEWL (black and green) and *Ac*NiR (red and blue) crystals loaded on the chip with either PolyPico or a pipette. Scaling and merging have been performed using *PRIME* on 12 546 and 13 563 integrated images for HEWL and AcNiR, respectively.

Importantly, the loading method does not significantly affect the quality of diffraction observed (Fig. 3 ▸). Both HEWL and *Ac*NiR crystals exhibit similar *R*_split_ and CC_1/2_ for both acoustic and pipette loading, and in all cases data quality is high. Differences in quality observed are of the same order as chip-to-chip variation when using the same loading approach, thus diffraction quality is not compromised by acoustic loading.

**Figure 4:**
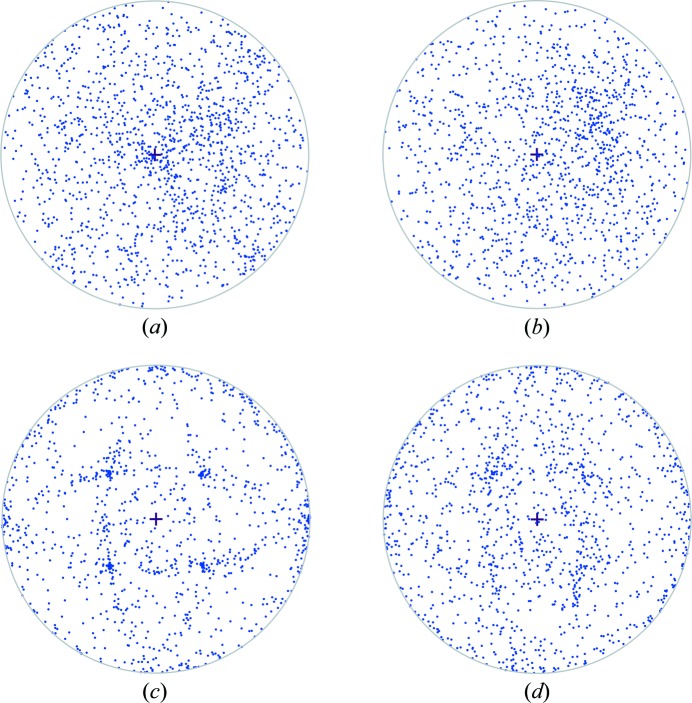
Stereographic projections illustrating the crystal orientation of 1000 randomly selected crystals for each loading method and crystal type: (*a*) pipette-loaded *Ac*NiR, (*b*) PolyPico-loaded *Ac*NiR, (*c*) pipette-loaded HEWL and (*d*) PolyPico-loaded HEWL. The plots represent the direction of the 001 *hkl* of each crystal (reindexed in *P*1) relative to the beam direction (*z*) which is shown as the central ‘+’ into the page. A point at 12 o’clock on the circular projection represents a 90° rotation of the crystal around *x* whereas the point at 3 o’clock represents a 90° rotation around *y.* Plots were produced using the module dials.stereographic_projection (Winter *et al.*, 2018 ▸).

For both pipette and acoustically loaded chips crystals are observed to be predominantly randomly orientated on the chips, illustrated by the stereographic projections in Fig. 4 ▸. To generate these plots data were reindexed in *P*1 so no symmetry equivalents are plotted. For more heavily loaded chips we do see some indication of systematic orientations, and this starts to become apparent in the case of pipette loaded HEWL [Fig. 4 ▸(*c*)]. Two orthogonal ellipses with a width of 70° at the centre of the projection become visible. These are consistent with loaded crystals lying on the internal walls of the chip apertures which are chemically etched along the silicon 111 crystal planes 54.74° from the surface of the chip (35.26° to the beam direction), with the ellipses reminiscent of stereographic projections of silicon etch planes as illustrated by Seidel *et al.* (1990 ▸). The degree of observed systematic orientation is likely to be dependent on the density of sample on the chip, crystal size and morphology, and also loading method, with acoustic loading less likely to yield systematic orientations.

## 4. Discussion   

Acoustic dispensing provides a means of reducing sample consumption for serial crystallography without compromising crystal quality with high-quality diffraction observed using both loading approaches. Acoustic dispensing has been previously exploited in the context of sample delivery, whereas here it is used for loading fixed targets that are subsequently passed to the beamline. This decoupling of acoustic ejection and X-ray data collection is advantageous as time taken to optimize drop ejection, which varies with the composition of the crystal slurry, does not impact the beam time efficiency.

Optimal loading is obtained with crystals less than ∼50 µm in size using cartridges with a 100 µm aperture. Increased hit-rates are obtained as the crystal slurry concentration increases, though settling of larger crystals or clumping can cause the PolyPico aperture to clog with time. This may explain why diffraction hit-rates do not increase as much as expected at the highest slurry concentration (Fig. 2 ▸). At lower crystal concentrations, we observe that the ejection process visibly disturbs the crystal slurry within the cartridge, slowing any settling process and multiple chips can be loaded from the same cartridge. Any long-term crystal settling can also be addressed by removing and reinserting the cartridge to resuspend the crystal slurry. In order to minimize any potential settling for high slurry concentrations, future loading setups will either make use of a rocking system or cartridges will be fed through a capillary fed by a syringe mounted on a rocker as used by Fuller *et al.* (2017 ▸).

While higher diffraction hit-rates can be obtained using traditional pipette loading, this is at the expense of increased sample consumption and the proportion of prepared crystals from which diffraction data are collected (*i.e.* the absolute hit-rate) falls. To obtain a similar number of indexed images, acoustic dispensing consumes tenfold less crystal slurry (*Ac*NiR, Fig. 2 ▸) than traditional pipette loading at the same sample concentration. Acoustic loading has the additional benefit that an increased fraction of the crystals produced for, and consumed by, the experiment result in diffraction. The success of acoustic loading is dependent on the chemical composition and viscosity of the crystal slurry and the parameters of the acoustic wave need to be optimized for each sample. As more viscous media may not be suitable for acoustic dispensing and the effect of crystal morphology is as yet unclear, acoustic loading of fixed targets is very much a complementary technique to pipette loading. We have demonstrated, however, that if samples are scarce, acoustic loading can help ensure a larger fraction of crystals see the X-ray beam and reduce the volume of sample required.

## Figures and Tables

**Table 1:** HEWL loading parameters and hit-rates The diffraction hit-rate (DHR) and absolute hit-rate (AHR) are defined in the text. Volumes dispensed (*V*) for the PolyPico are an upper bound: droplet volumes vary from drop to drop so a conservative average value is used.

Loading method	Crystal concentration (crystals ml^−1^)	*V*(µl)	Calculated crystals dispensed	Indexed patterns	DHR (%)	AHR (%)
Pipette	5 × 10^4^	75	3750	2053	14.3	55
Pipette	1 × 10^5^	75	7500	2129	14.8	28
Pipette	5 × 10^5^	75	37500	4850	33.7	13
Pipette	1 × 10^6^	75	75000	10462	72.6	14
PolyPico	5 × 10^5^	3	1500	1311	9.1	87
PolyPico	1 × 10^6^	3	3000	1763	12.2	59
PolyPico	1.5 × 10^6^	3	4500	2883	20.0	64
PolyPico	2 × 10^6^	3	6000	4573	31.8	76
PolyPico	2.5 × 10^6^	3	7500	3355	23.3	45

**Table 2:** *Ac*NiR loading parameters and hit-rates The diffraction hit-rate (DHR) and absolute hit-rate (AHR) are defined in the text; *V* is the volume dispensed. Note that for pipette-loaded *Ac*NiR, diffraction data were collected from a full chip (25 600 apertures) in contrast to all other data which were collected from 14 400 apertures.

Loading method	Crystal concentration (crystals ml^−1^)	*V* (µl)	Calculated crystals dispensed	Indexed patterns	DHR (%)	AHR (%)
Pipette	1.2 × 10^5^	150	18000	1185	4.6	6.6
Pipette	1.8 × 10^5^	150	27000	1687	6.6	6.2
Pipette	2.5 × 10^5^	150	37500	3574	14.0	9.5
Pipette	3.7 × 10^5^	150	55500	4145	16.2	7.5
Pipette	8.5 × 10^5^	150	127500	6223	24.3	4.9
Pipette	1.7 × 10^6^	150	255000	6587	25.7	2.6
Pipette	3.7 × 10^6^	150	555000	13061	51.0	2.4
PolyPico	4.3 × 10^5^	3	1290	332	2.3	25.7
PolyPico	8.6 × 10^5^	3	2580	878	6.1	34.0
PolyPico	1.7 × 10^6^	3	5100	2023	14.1	39.7
PolyPico	2.2 × 10^6^	3	6600	1979	13.7	30.0
PolyPico	4.4 × 10^6^	3	13200	2526	17.5	19.1
